# Differences in Cortical Surface Area in Developmental Language Disorder

**DOI:** 10.1162/nol_a_00127

**Published:** 2024-06-03

**Authors:** Nilgoun Bahar, Gabriel J. Cler, Saloni Krishnan, Salomi S. Asaridou, Harriet J. Smith, Hanna E. Willis, Máiréad P. Healy, Kate E. Watkins

**Affiliations:** Department of Experimental Psychology & Wellcome Trust Centre for Integrative Neuroimaging, University of Oxford, Oxford, UK; Department of Speech & Hearing Sciences, University of Washington, Seattle, WA, USA; Department of Psychology, Royal Holloway, University of London, Egham Hill, Surrey, UK; MRC Cognition & Brain Sciences Unit, University of Cambridge, Cambridge, UK; Nuffield Department of Clinical Neuroscience, University of Oxford, Oxford, UK; Department of Psychology, University of Cambridge, Cambridge, UK

**Keywords:** developmental language disorder, paediatric, structural asymmetry, structural MRI, surface area, surface-based anatomical modeling

## Abstract

Approximately 7% of children have developmental language disorder (DLD), a neurodevelopmental condition associated with persistent language learning difficulties without a known cause. Our understanding of the neurobiological basis of DLD is limited. Here, we used FreeSurfer to investigate cortical surface area and thickness in a large cohort of 156 children and adolescents aged 10–16 years with a range of language abilities, including 54 with DLD, 28 with a history of speech-language difficulties who did not meet criteria for DLD, and 74 age-matched controls with typical language development (TD). We also examined cortical asymmetries in DLD using an automated surface-based technique. Relative to the TD group, those with DLD showed smaller surface area bilaterally in the inferior frontal gyrus extending to the anterior insula, in the posterior temporal and ventral occipito-temporal cortex, and in portions of the anterior cingulate and superior frontal cortex. Analysis of the whole cohort using a language proficiency factor revealed that language ability correlated positively with surface area in similar regions. There were no differences in cortical thickness, nor in asymmetry of these cortical metrics between TD and DLD. This study highlights the importance of distinguishing between surface area and cortical thickness in investigating the brain basis of neurodevelopmental disorders and suggests the development of cortical surface area to be of importance to DLD. Future longitudinal studies are required to understand the developmental trajectory of these cortical differences in DLD and how they relate to language maturation.

## INTRODUCTION

Developmental language disorder (DLD) is a neurodevelopmental condition that affects approximately 7.5% of children (Norbury et al., 2016). Children with DLD have persistent difficulties in developing age-appropriate receptive and expressive language skills (Bishop, 2017) and are at increased risk of behavioural, social, academic, and later vocational problems (e.g., Conti-Ramsden et al., 2018; Goh et al., 2021). Importantly, these language deficits cannot be attributed to known biomedical or environmental causes, such as brain injury, neurological conditions (e.g., epilepsy), childhood hearing loss, or social disadvantage (Bishop et al., 2017). Our understanding of the neurobiological basis of DLD remains limited. Heritability studies and genome screening in DLD provide evidence that the disorder is linked to genetic variation (see review by Mountford et al., 2022). These subtle disruptions most likely affect the development of neural circuits involved in language acquisition at pre- and postnatal developmental stages (den Hoed & Fisher, 2020; Newbury & Monaco, 2010). Using advances in analysis of structural brain imaging data, we are increasingly sensitive to detecting these neural differences and identifying potential endophenotypes linking behavioural, neural, and genetic variation in DLD. Here, we used analysis of brain images obtained in a large, well-characterised cohort of children with a range of language learning abilities, including DLD and typically developing (TD) controls, to explore differences in cortical morphometry that could be related to differences in language learning.

The existing literature on the neuroanatomical correlates of DLD has relied on manual tracing or semiautomated methods to study the size, shape, or asymmetry of cortical and subcortical structures as well as white matter microstructure (see Asaridou & Watkins, 2022, for an overview). Some studies have also examined whether variation in neuroanatomical structures relates to performance on neuropsychological measurements (e.g., Badcock et al., 2012; Kurth et al., 2018; Soriano-Mas et al., 2009). Cumulative evidence indicates a distributed, bilateral pattern of subtle morphological abnormalities focused on perisylvian cortical areas involved in speech and language, and subcortical structures implicated in habit formation and sequential learning (see Mayes et al., 2015, for a review). However, findings are inconsistent among these studies in terms of the location and direction of differences (i.e., whether regions are larger or smaller in DLD) possibly due to different methods used, small sample sizes, and the age ranges of different cohorts (see Table 1 for details).

**Table 1.** Summary of studies investigating structural grey matter correlates of DLD

Study	Participants		Method	Results
	Group (*n*)	Mean age in years		
Lee et al. (2020)	Cohort 1		FreeSurfer ROI analysis	➢ DLD > TD − L transverse temporal gyrus; L & R temporal poles; R pars orbitalis.
	DLD (14)	22.42		
	TD (12)	22.1		
	Cohort 2			
	DLD (19)	16.96		
	TD (61)	16.62		
Pigdon et al. (2019)	DLD (17)	9–11	Whole-brain & ROI VBM	➢ DLD > TD − R cerebellum. Also in R inferior frontal gyrus, posterior superior temporal gyrus, putamen but these did not survive Bonferroni correction. ➢ Uncorrected comparisons at *p* < 0.001 showed larger peak volume in transverse temporal gyrus, pars triangularis, parahippocampal gyrus, and caudate tail on the right side in DLD, less peak volume in the R cuneus and inferior occipital lobe.
	TD (40)			
Kurth et al. (2018)	DLD (13)	10.21	VBM of ROIs based on cytoarchitectonic atlas	➢ DLD > TD − R pars triangularis. ➢ Greater R > L asymmetry in DLD (pars triangularis) at trend level.
	TD (18)	10.2		
Girbau-Massana et al. (2014)	SLI (14)	10.08	Whole-brain VBM	➢ TD > SLI − L & R medial occipital gyri; R postcentral gyrus. ➢ SLI > TD − R superior occipital gyrus.
	TD (10)	9.33		
Lee et al. (2013)	DLI (12)	21.99	ROI analysis with AutoWorkup	➢ DLI > TD − L & R putamen & nucleus accumbens; R globus pallidus.
	TD (12)	22.06		
Badcock et al. (2012)	SLI (10)	13.5	Whole-brain VBM	➢ SLI > TD − L inferior frontal gyrus; L intraparietal sulcus; R insula. ➢ TD > SLI − Posterior superior temporal sulcus bilaterally; R superior temporal gyrus; R caudate nucleus; R substantia nigra; medial frontal polar cortex; R medial superior parietal cortex; L occipital pole.
	TD (16)	12.5		
Soriano-Mas et al. (2009)	Whole sample (5–17)		Whole-brain VBM	➢ SLI > TD − Mean global volume; R posterior superior temporal gyrus; L middle occipital gyrus. ➢ Positive correlation (age & grey matter volume) in TD but not SLI.
	SLI (36)	10.58		
	TD (36)	10.88		
	Younger cohort			➢ SLI > TD − Mean global volume; entorhinal area bilaterally; L temporopolar cortex; L caudate nucleus; L motor cortex; L precuneus. ➢ Increase with age in several of these regions in TD but not SLI.
	SLI (19)	<11		
	TD (14)			
	Older cohort			➢ No group differences.
	SLI (17)	>11		
	TD (22)			
Jäncke et al. (2007)	DLD (21)	8.33 (4–10)	Whole-brain VBM	➢ No group differences in grey matter volume. See paper for group differences in white matter volume.
	TD (21)	9.26 (4–10)		
Herbert et al. (2005)	DLD (15)	5.7–11.3	Whole-brain manual & semiautomated parcellation/segmentation	➢ Higher aggregate cortical parcellation unit asymmetry in DLD Higher R > L asymmetry in DLD. ➢ Lower L > R asymmetry in DLD. ➢ Group differences in unimodal & higher-order association cortex but not in primary sensory-motor cortex.
	TD (15)			
De Fossé et al. (2004)	SLI (9)	9.9	ROI manual & semiautomated parcellation/segmentation	➢ Atypical asymmetry in language-related brain regions in SLI. ➢ Reduced L > R volumetric asymmetry of Broca’s area in SLI. ➢ Reversed (R > L) asymmetry in inferior frontal gyrus in SLI. ➢ Positive correlation between verbal IQ & greater leftward pars triangularis asymmetry in SLI.
	TD (11)	10.4		
Herbert et al. (2003)	DLD (24)	8.24	Whole-brain manual & semiautomated analysis of major brain regions	➢ DLD > TD in total brain size, driven by increase in cerebral white matter volume. ➢ Cerebral cortex & caudate nucleus relatively but not absolutely smaller in DLD.
	TD (30)	9.13		
Preis et al. (1998)	DLD (21)	8.33	ROI manual parcellation	➢ No difference in planum temporale & planum parietale asymmetry. ➢ Planum temporale bilaterally smaller in DLD (accounted for by smaller forebrain).
	TD (21)	8.25		
Gauger et al. (1997)	SLI (11)	9.1	Manual ROI	➢ TD > SLI − L pars triangularis. ➢ Reduced L > R asymmetry (pars triangularis, planum temporale, posterior ascending ramus).
	TD (19)	8.9		
Plante et al. (1991)	SLI (8)	4.17–9.5	Manual ROI	➢ Atypical (R = L & R > L) asymmetry of perisylvian areas in SLI compared to TD. ➢ R perisylvian areas significantly larger in SLI.
	TD (8)			
Jernigan et al. (1991)	LI (20)	8.9	Whole-brain semiautomated & manual morphometry	➢ TD > LI − L posterior perisylvian region. ➢ Less L/R ratio in LI (prefrontal cortex inferior to plane above frontal operculum, including orbitofrontal, dorsolateral, & medial frontal cortex). ➢ More L/R ratio in LI (superior parietal lobe above parietal operculum; small portion of the most superior occipital lobe).
	TD (12)	9.0		

*Note*. The second column provides information of the sample size (only for DLD and controls) and their mean age in years. Results pertain to grey matter volume measurements of cortical regions, unless otherwise specified.

*Abbreviations*: DLD = developmental language disorder; TD = typically developing; ROI = region of interest; VBM = voxel-based morphometry; SLI = specific language impairment; DLI = developmental language impairment; LI = language or learning-impaired children; R = right; L = left.

In terms of cortical anomalies in the frontal lobes, both smaller (Gauger et al., 1997) and larger (Badcock et al., 2012) size of the left inferior frontal gyrus in DLD were reported. Greater volume in DLD was also found in perisylvian regions in the right hemisphere (Plante et al., 1991; Soriano-Mas et al., 2009) including in the pars orbitalis (Lee et al., 2020), pars triangularis (Kurth et al., 2018), and the insula (Badcock et al., 2012). Posteriorly, there are reports of lower volume in DLD in the left (Jernigan et al., 1991) as well as the right hemisphere, comprising the planum temporale (Preis et al., 1998), other portions of the superior and middle temporal gyri, and parietal regions (Badcock et al., 2012; Girbau-Massana et al., 2014). Greater volume in DLD was seen in the transverse temporal gyrus, temporopolar cortex (Lee et al., 2020; Soriano-Mas et al., 2009), medial parietal cortex (Soriano-Mas et al., 2009), and intraparietal sulcus (Badcock et al., 2012). Smaller volume of the right perisylvian region and the occipital petalia were associated with higher verbal intelligence and receptive vocabulary in DLD, respectively (Soriano-Mas et al., 2009). Some studies did not find grey matter differences between DLD and TD (e.g., Jäncke et al., 2007).

Another focus of brain imaging studies in DLD explores whether atypical structural asymmetries could provide clues to the neuroanatomical basis of functional lateralisation in DLD. The idea of atypical functional laterality is a long-standing theoretical attempt to explain speech and language difficulties in neurodevelopmental disorders including DLD, in which the language difficulties are attributed to a failure to establish typical lateralisation patterns for speech and language (see Bishop, 1990, 2013, for review). The evidence from the task-based imaging literature to support this theory remains mixed, however. Badcock and colleagues (2012) found brain activity evoked during a covert naming task to be less left lateralised in children with DLD compared with their siblings and children in the TD group. Similarly, de Guibert et al. (2011) found a lack of left lateralisation in DLD across single or combined tasks in all frontal and temporoparietal language regions of interest (ROIs). In contrast, many studies failed to provide support for this hypothesis (Dibbets et al., 2006; Hugdahl et al., 2004; Krishnan et al., 2021; Weismer et al., 2005).

The same pattern of mixed findings dominates research on the asymmetry of brain structures. Atypical structural asymmetry in perisylvian areas was reported in DLD, especially less leftward asymmetry of the inferior frontal gyrus (see Mayes et al., 2015, for a review). More pronounced rightward (i.e., right-greater-than-left) asymmetry of pars triangularis was reported in DLD, and a positive correlation showing that higher verbal intelligence quotient (IQ) scores were associated with more leftward asymmetry of pars triangularis (De Fossé et al., 2004; Kurth et al., 2018). The atypical pars triangularis asymmetry was specifically associated with language difficulties; boys with DLD or with autism spectrum disorder (ASD) and language impairment showed atypical asymmetry, whereas those with ASD but no language impairment did not (De Fossé et al., 2004). In a whole brain analysis, children with DLD showed greater rightward asymmetry in the aggregate amount of cortical parcellation units and smaller volume of left-asymmetrical cortex, with significant differences in language-related cortical parcellations, including the posterior supramarginal gyrus and planum polare (Herbert et al., 2005). Another study found atypical (right-greater-than-left) asymmetry in the prefrontal region and left-greater-than-right asymmetry in the parietal region in language impaired children, but no group differences in the posterior perisylvian regions (Jernigan et al., 1991). A study of a small sample of boys with DLD found atypical perisylvian asymmetries due to larger right hemisphere structures, while the left structures were of typical size (Plante et al., 1991). In contrast, typical asymmetry of the planum temporale and planum parietale was reported for a cohort of children with DLD and their age-matched controls (Preis et al., 1998).

An important limitation in past studies of brain morphology in DLD is that almost all findings pertain to measures of volume, which in the cortex depends on both surface area and cortical thickness. It remains unclear whether the reported cortical morphological abnormalities reflect differences in surface area, cortical thickness, or both. Surface area and cortical thickness are heritable anatomical features that are under separate genetic influences and have independent developmental trajectories, suggesting that their changes during childhood reflect different developmental processes (Panizzon et al., 2009; Winkler et al., 2010). Early in brain development, cortical surface area and thickness are thought to depend on pre- and early post-natal processes of neurogenesis, including the number of radial units, cell proliferation, neuronal migration, programmed cell death, and the density of the columns themselves (Rakic, 2009; Tamnes et al., 2017). In addition, surface area is closely linked to cortical folding, which in turn depends on the division of progenitor cells during early embryonic stages (Chenn & Walsh, 2002; Rakic, 2009). Surface area expansion during childhood is most likely driven by increases in dendritic complexity, spine and synaptic density, and glial cell numbers, along with axonal growth and intracortical myelination of these axons. Cortical thickness reductions (or “thinning”) may be due to a decrease in synaptic density that arises from experience-dependent plasticity (Rakic, 2009; Sowell et al., 2004), and increased intracortical myelin potentially “whitening” the cortex (Paus et al., 2008). Investigating surface area and cortical thickness as distinct grey matter volume components may therefore enhance our understanding of the neurogenetic pathways underlying language learning difficulties in children with DLD.

Here, we addressed the shortcomings of previous studies by investigating differences in cortical surface area and thickness in a large and well-characterised sample of children with DLD compared with a group of TD controls. FreeSurfer (Fischl, 2012) was used to generate mesh models of the cerebral cortex from magnetic resonance imaging (MRI) scans. We also investigated asymmetries in these measures using a reproducible and automated surface-based interhemispheric registration method (see Greve et al., 2013; Sha et al., 2021). Based on the previous literature and behavioural profile, we predicted that relative to TD controls, children with DLD would exhibit: (1) differences in the morphology of perisylvian regions relevant for language, specifically the inferior frontal gyrus; and (2) atypical cortical asymmetry between the same left hemisphere regions and their right hemisphere homologues. We expected morphological differences to include right hemisphere homologues of language regions due to accounts of children with typical language acquisition following focal unilateral brain injury (e.g., Newport et al., 2017), or even without a left hemisphere (Asaridou et al., 2020; Curtiss et al., 2001), indicating the potential of the intact right hemisphere to support language. Accordingly, in DLD where there are no obvious lesions to explain the language impairment, we expect that causal abnormalities in the cortex would occur bilaterally, else the high degree of plasticity available during development would support reorganisation of function to the unaffected hemisphere. Furthermore, given DLD’s genetic component (Bishop, 2014), it is likely that genetic disruptions will affect neural development of pathways in both hemispheres rather than only one (e.g., Belton et al., 2003), though the latter possibility cannot be ruled out.

## MATERIALS AND METHODS

### Ethics

Ethical approval was granted by the Medical Sciences Interdivisional Research Ethics Committee at the University of Oxford (R55835/RE002). We obtained written informed consent from the parents/guardians of the participants and written assent from the participants.

### Participant Recruitment and Screening

One hundred and seventy-five children took part in the Oxford Brain Organisation in Language Development (OxBOLD) project (see Krishnan et al., 2021, 2022). We included participants older than 10 years and younger than 16 who had grown up in the United Kingdom speaking English. Participants were recruited through a range of venues, including those who took part in previous studies: the SCALES study (Norbury et al., 2016), the Wellcome Reading and Language Project (Snowling et al., 2016), and the OSCCI Twins study (Wilson & Bishop, 2018). We also recruited from schools for children with language learning difficulties and organisations conducting outreach with those with language impairments (ICAN, Afasic, RADLD), as well as dyslexia (British Dyslexia Association). Typically developing controls were recruited mainly from local schools and schools participating in university outreach programs.

Parents of the participants completed the Strengths and Difficulties Questionnaire (SDQ; Goodman, 1997) and the Social Communication Questionnaire (SCQ; Rutter et al., 2003). Participants were not recruited if they (1) had a diagnosis of another developmental disorder (e.g., Down syndrome, ASD, or ADHD); (2) indicated a history of neurological impairments or neurological disorders such as epilepsy; (3) scored above seven on the Hyperactivity subscale of the SDQ (Goodman, 1997); (4) scored above 15 on the SCQ (Rutter et al., 2003); or (5) had any contraindication to MRI. We did not exclude participants based on handedness or speaking multiple languages.

### Behavioural Measures

Parents of participants completed an intake questionnaire on general demographic information (e.g., age, gender, language background, parental education, medical history). Socioeconomic status was estimated based on maternal education levels, which were categorised into eight subgroups based on the United Kingdom’s Regulated Qualifications Framework (https://www.gov.uk/what-different-qualification-levels-mean/list-of-qualification-levels). We subsequently collapsed the eight categories into five ranks (1–2 [left education aged ∼16 yr] = 1; 3–5 [stayed in education to age ∼18 yr] = 2; 6 [bachelor’s degree or equivalent] = 3; 7 [master’s degree] = 4; 8 [doctorate] = 5).

Parents also completed the Children’s Communication Checklist—Version 2 (CCC-2; Bishop, 2003a), a screening questionnaire used to facilitate the identification of social communication difficulties and their potential impact on daily life in children with language disorder.

Each participant was assessed using a comprehensive neuropsychological test battery (lasting approximately 2 hr) to evaluate their hearing, language, memory, nonverbal reasoning, motor, and reading skills.

#### Language

Language skills were assessed through five standardised tests which focused on vocabulary, grammar, and narrative skills in both the receptive and expressive domains. We used the Receptive One-Word Picture Vocabulary—Fourth Edition (ROWPVT-4; Martin & Brownell, 2011) and Expressive One-Word Picture Vocabulary—Fourth Edition (EOWPVT-4; Martin & Brownell, 2010) to test children’s receptive and expressive vocabulary, respectively. Comprehension of grammar was assessed using the Test for Reception of Grammar—Second Edition (Bishop, 2003b). We used the Recalling Sentences subtest of the Clinical Evaluation of Language Fundamentals—Fourth Edition (Semel et al., 2003) as a proxy for expressive grammar. Narrative skills were assessed using the Expression, Reception, and Recall of Narrative Instrument (Bishop, 2004), which provides measures of story comprehension and the child’s ability to recall it initially and after a short delay. All scores obtained in this section were converted into age-scaled scores using published norms. Six scores from these five language tests were used to categorise the children into groups (see below); one each from expressive and receptive vocabulary and grammar, the comprehension score from the narrative test and either the initial or the delay recall score but not both.

We also administered a nonword repetition test (Norbury et al., 2016; Snowling et al., 2016). Only raw scores are reported as this test was not norm-referenced.

#### Memory

The Digit Span Forward and Digit Span Backward subtests from Children’s Memory Scale (CMS; Cohen, 1997) were used to assess the participants’ auditory short-term and working memory, respectively. We also administered the Word Lists subtest from the CMS to investigate episodic long-term memory. Raw scores from all CMS subtests were converted into age-scaled scores using published norms.

#### Nonverbal reasoning

The Block Design, Matrix Reasoning, and Coding subtests of the Wechsler Intelligence Scale for Children—Fourth Edition (WISC-IV; Wechsler, 2003) were administered to estimate nonverbal reasoning skills. The results obtained from the first two subtests were used to calculate a mean composite nonverbal IQ score.

#### Reading

The Test of Word Reading Efficiency—Second Edition (TOWRE-2; Torgesen et al., 1999) was used to assess word (Sight Word Efficiency) and nonword decoding (Phonemic Decoding Efficiency) skills.

#### Motor

We used the Oromotor Sequences subtest of the NEuroPSYchology test (NEPSY; Korkman et al., 1998) to assess the participants’ oromotor coordination. We estimated handedness based on the participants’ self-reported preferred hand for writing. Relative manual dexterity was assessed through two subtests of the Purdue Pegboard Test (PPT; Brookman et al., 2013; Tiffin, 1968). These scores were age- and gender-scaled based on the PPT manual’s published norms.

### Study Sample

All children passed audiometric screening at 25 dB at 500 Hz, 1000 Hz, and 2000 Hz in the better ear. After testing, we excluded data from 15 children for the following reasons: three obtained a mean nonverbal IQ below 70 (2 standard deviations below the mean); three were later discovered to have not grown up in the United Kingdom speaking English; one participant had scores on two or more tests of language that were more than 1 standard deviation below the mean but no history of speech and language difficulties; two did not complete the behavioural assessment; three did not have MRI; three were excluded due to incidental findings of unknown clinical significance on the MRI. These exclusions resulted in an initial cohort of 160 children for whom both MRI and behavioural data were available for analysis.

Consistent with previous studies (Hsu & Bishop, 2014; Tomblin et al., 1996; see Bishop et al., 2016, for discussion), children were categorised as having DLD if they (1) had a previously reported history of speech-language difficulties, and (2) received a score that was one or more standard deviations below the normative mean on two or more of the six scores from standardised language tests administered (see above). Participants were categorised as TD if they had no history of speech and language problems and had no more than one language test score of one standard deviation or more below the normative mean.

Out of the 160 participants, 55 met the criteria for DLD (*m* age = 12.36 years; 15 female), and 77 were classified as TD (*m* age = 12.52 yr; 33 female). An additional 28 did not meet the criteria for DLD at the time of testing but had a history of speech-language problems (HSL; *m* age = 12.39 yr; 5 female).

In addition to analyses comparing TD and DLD groups on the brain measures, a language factor was used in continuous analyses of the whole cohort including the HSL group. We used factor analysis to derive this language factor based on the best weighted combination of the participants’ language and memory measures described above (see Krishnan et al., 2021, for details).

### MRI Acquisition

Brain scans were acquired on a 3-T Siemens Prisma scanner with a 32-channel radiofrequency head coil. The anatomical images were obtained in the sagittal plane using a T1-weighted 3-D MPRAGE sequence with the following pulse sequence parameters: repetition time = 1,900 ms, echo time = 3.97 ms, flip angle = 8°, inversion time = 904 ms, with 192 × 192 sagittal field of view, 174 slices, and voxel size = 1 × 1 × 1 mm. Participants wore earplugs and noise-cancelling headphones held in place by inflatable air pads. The total image acquisition time was 5 min and 30 s.

### MRI Processing

Prior to processing, an experienced rater carefully checked structural scans for artefacts. We used FreeSurfer (Version 7.2.0; https://surfer.nmr.mgh.harvard.edu) to automatically parcellate T1-weighted images and generate 3-D reconstructions of each participant’s cortex via the “recon-all” command (Dale et al., 1999; Fischl et al., 1999). The reconstructed surfaces were then registered to the *fsaverage* template and assigned a neuroanatomical label (Fischl et al., 1999). To label the sulci and gyri, the Desikan-Killiany atlas was employed, which consists of 33 ROIs per hemisphere, for a total of 66 ROIs (Desikan et al., 2006; Fischl et al., 2004).

We used the Qoala-T supervised-learning tool (Klapwijk et al., 2019) to assess the quality of cortical segmentations and parcellations post-processing. This automated approach provided a number of advantages over manual correction, including reduced time and variability associated with manual quality control (e.g., inter-rater bias). Four participants were excluded after running Qoala-T (Klapwijk et al., 2019) on the T1-weighted images. Qoala-T evaluates scan quality on a 0–100 scale. Nine scans with an average scan quality score below 50 were recommended for exclusion (the mean Qoala−T score of our entire dataset was 71.5). We retained five of these scans after visually inspecting the parcellation qualities. We additionally inspected the quality of image segmentations and cortical reconstructions in 51 scans with average quality above 50, as recommended by Qoala-T. We retained all 51 scans. The final cohort consisted of 54 children with DLD (*m* age = 12.43 yr; 15 female), 74 TD (*m* age = 12.6 yr; 33 female), and 28 HSL (*m* age = 12.4 yr; 5 female).

### Cortical Metrics

Our morphometric measures of interest were surface area, cortical thickness, and grey matter volume. Surface area is defined as the average area of the triangles (on the tessellated surface) of which each vertex is a member, while cortical thickness is the distance between the white and pial surfaces at each vertex (Fischl & Dale, 2000). Grey matter volume is the product of surface area multiplied by cortical thickness at each vertex.

### Whole-Brain Cortical Morphometry

The surface area, cortical thickness, and grey matter volume maps were spatially smoothed in each hemisphere using a Gaussian smoothing kernel with a full width at half maximum of 10 mm. A two-group general linear model (GLM) was performed to investigate cortical differences between DLD and TD in vertex-wise whole-brain analyses. Cortical area, thickness, and volume were separately entered as outcome variables with participants’ age and sex as covariates of no interest. Clusters were defined as groups of contiguous vertices using a vertex-wise threshold of *p* < 0.001 (two-tailed). We corrected for multiple comparisons by using a cluster-wise correction with a Monte Carlo simulation of 10,000 iterations and a threshold of *p* < 0.05 (two-tailed).

Children in the HSL group were excluded from the whole-brain DLD–TD comparisons. Instead, we included them in two continuous analyses: first, to examine the effects of age and sex and their interaction on surface area, thickness, and volume at each vertex across all participants; and second to investigate the relationship between language ability and the cortical metrics across the whole cohort. For the latter, we used the constructed language factor scores (see Study Sample) in a GLM controlling for age and sex. The same smoothing kernel and correction for multiple comparisons method described above was used in both these analyses.

### Surface-Based Structural Asymmetry Analysis

Following the steps proposed by Greve and colleagues (2013), we first registered the participants’ surface area, cortical thickness, and grey matter volume maps of both hemispheres to FreeSurfer’s symmetric standard template (*fsaverage_sym*). This step aimed to achieve vertex-wise correspondence between the two hemispheres through precise alignment of the cortical folding patterns between- and within-participants. For each participant, we calculated the asymmetry quotient (AQ) separately for each pair of vertices matched across the left (L) and right (R) hemispheres using the equation AQ = [L − R] / [(L + R)/2], with negative values indicating right- and positive values indicating left-lateralisation. We masked out a region around the boundary between cortical and subcortical structures which had extreme AQ values in many participants, likely due to a warping artefact. We excluded any vertices from all individuals with asymmetry values of |AQ| > 1 in at least 25% (*n* = 31) of our participants (see Figure 1 of the Supplementary Material, available at https://doi.org/10.1162/nol_a_00127). Vertices with extreme AQ values were subsequently excluded from the thickness and volume analyses.

We used the vertex-wise AQ values between the DLD–TD participants as the dependent variable in a GLM with age and sex as covariates of no interest. Given previous reports of sex differences in grey matter in Broca’s area (Kurth et al., 2017), we also assessed the interaction between group and sex. AQ values from surface area, thickness, and volume were each entered into the model above. Significant clusters were determined using SurfStat (Worsley, 2008) with a cluster-forming threshold of *p* < 0.05 (vertex-wise *p* < 0.001) and assessed against a random field with the same spatial correlation as the real map to minimise the likelihood of false positives. Clusters identified in this manner had a peak vertex and contiguous vertices, with cluster-wise significance determined.

## RESULTS

### Demographic and Behavioural Data

Table 2 presents the demographic information and results from the neuropsychological battery for the DLD, TD and HSL groups. There were no significant differences among groups in mean age, distribution of handedness, maternal education levels, or in number of other languages spoken at home. While the TD group was balanced for sex, there were more males than females in the DLD and HSL groups, and these ratios differed significantly from the TD ratio (*χ*^2^ (2, *N* = 156) = 7.88, *p* = 0.019) but not from each other. The DLD group scored significantly lower than TD on all measures of language, memory, nonverbal reasoning, motor, and reading skills. Results from the CCC-2 (Bishop, 2003a) revealed that the TD and DLD groups also differed in their General Communication Composite (GCC) values, with the latter having significantly lower structural language scores. The HSL group also had significantly lower GCC scores than the TD group, but these were significantly higher than the DLD group.

**Table 2.** Demographic and behavioural data for the TD, DLD, and HSL groups

	TD (*n* = 74)	DLD (*n* = 54)	HSL (*n* = 28)	*p* value	Group differences
**Demographics**					
Mean age (years)	12.6 ± 0.2	12.4 ± 0.3	12.4 ± 0.3	0.785	–
Age range (years;months)	10;0–15;7	10:0–15;11	10;5–15;7	–	–
Sex (M:F)	41:33	39:15	23:5	**0.019**	TD < DLD, HSL
Handedness (R:L)	63:11	46:8	24:4	0.997	–
Language(s) spoken at home other than English (Yes)	13.43% (*n* = 67)	5.55%	10.71%	0.39	–
Maternal education (range)	3 (1–5) (*n* = 69)	3 (1–5)	3 (2–4)	0.065	–
**Language**					
ROWPVT-4	128.4 ± 1.9	100.1 ± 2.1	122 ± 2.9	**<0.001**	DLD < TD, HSL
EOWPVT-4	117.9 ± 1.7	92.4 ± 1.7	108.3 ± 2.5	**<0.001**	DLD < HSL < TD
TROG-2	105.2 ± 0.9 (*n* = 73)	80.9 ± 1.8 (*n* = 53)	98.9 ± 1.6	**<0.001**	DLD < HSL < TD
CELF-4 Recalling Sentences^a^	11.6 ± 0.3 (*n* = 70)	4.9 ± 0.4	9 ± 0.6	**<0.001**	DLD < HSL < TD
ERRNI					
Initial Recall	100.1 ± 1.5	83.7 ± 1.7	96.3 ± 2.1	**<0.001**	DLD < TD, HSL
Delayed Recall	104.4 ± 1.3	85.1 ± 1.7	99.1 ± 1.9	**<0.001**	DLD < TD, HSL
Comprehension	106.7 ± 1.6	93.2 ± 2.1	101.9 ± 1.9	**<0.001**	DLD < TD, HSL
Nonword repetition (max. 30)^b^	26.3 ± 0.3 (*n* = 71)	17.8 ± 0.8 (*n* = 53)	22.6 ± 0.7	**<0.001**	DLD < HSL < TD
**Memory**					
CMS					
Digit Span Forward^a^	11.5 ± 0.3	5.8 ± 0.4	7.5 ± 0.6	**<0.001**	DLD, HSL < TD
Digit Span Backward^a^	11.8 ± 0.3	7.2 ± 0.5	8.5 ± 0.6	**<0.001**	DLD, HSL < TD
Word List Immediate Recall^a^	10.1 ± 0.4	6.2 ± 0.4 (*n* = 52)	8.4 ± 0.5	**<0.001**	DLD < HSL < TD
Word List Delayed Recall^a^	10.2 ± 0.4 (*n* = 73)	7.3 ± 0.5 (*n* = 51)	9.8 ± 0.6	**<0.001**	DLD < TD, HSL
Word List Delayed Recognition^a^	8.6 ± 0.4 (*n* = 73)	6.6 ± 0.5	7.4 ± 0.6	**0.003**	DLD, HSL < TD
**Nonverbal reasoning**					
WISC-IV					
Block Design^a^	13.3 ± 0.2	9.8 ± 0.4	12.8 ± 0.4	**<0.001**	DLD < TD, HSL
Matrix Reasoning^a^	11.2 ± 0.3	7.6 ± 0.4	9.9 ± 0.5	**<0.001**	DLD < TD, HSL
Coding^a^	9.6 ± 0.3 (*n* = 71)	5.9 ± 0.4	6.5 ± 0.4	**<0.001**	DLD, HSL < TD
**Reading**					
TOWRE-2					
Sight Word Efficiency	106 ± 1.3	82.6 ± 1.9	89.1 ± 2.6	**<0.001**	DLD, HSL < TD
Phonemic Decoding Efficiency	112.2 ± 1.6	81.3 ± 2.2	87.1 ± 2.5	**<0.001**	DLD, HSL < TD
**Motor**					
NEPSY Oromotor Sequencing (max. 70)^b^	60.5 ± 0.9	41.4 ± 1.5	53.4 ± 1.9	**<0.001**	DLD < HSL < TD
Purdue Pegboard					
Pegs moved with dominant hand (z-score)^b^	−0.4 ± 0.1	−1.5 ± 0.2	−0.9 ± 0.2	**<0.001**	DLD < HSL < TD
Pegs moved with non-dominant hand (z-score)^b^	0.0 ± 0.1	−1.1 ± 0.2	−0.8 ± 0.2	**<0.001**	DLD < HSL < TD
Mean difference (dominant − non-dominant)^b^	0.6 ± 0.1	0.6 ± 0.2	0.6 ± 0.2	**<0.001**	DLD < HSL < TD
**CCC-2**					
GCC^c^	85.3 ± 1.7	35.9 ± 2.7 (*n* = 53)	48.4 ± 4.4	**<0.001**	DLD < HSL < TD

*Note*. Means along with the standard error of the mean (± SEM) are provided unless otherwise stated. Sex, handedness ratios, and the results on the language background questionnaire (answered yes/no) were compared with a chi-squared test. Maternal education level was compared using the Kruskal–Wallis test, and the median for each group (ranked 1–5; 3 = degree-level education) is presented (with the range in parentheses). All other variables were compared between groups using analysis of variance, with post-hoc comparisons using Tukey’s method. The *p* values of these group differences are displayed. The last two columns display the *p* values (with those with *p* < 0.05 shown in **bold** typeface) and whether there were significant group differences when using *t* tests or chi-squares (*p* < 0.05). Tests without a superscript denote standard scores with a standard mean of 100 and SD of 15.

*Abbreviations*: TD = typically developing; DLD = developmental language disorder; HSL = history of speech-language difficulties; ROWPVT-4 = Receptive One-Word Picture Vocabulary—Fourth Edition; EOWPVT-4 = Expressive One-Word Picture Vocabulary—Fourth Edition; TROG = Test for Reception of Grammar–Version 2; CELF-4 = Clinical Evaluation of Language Fundamentals—Fourth Edition; ERRNI = Expression, Reception and Recall of Narrative Instrument; CMS = Children’s Memory Scale; WISC-IV = Wechsler Intelligence Scale for Children—Fourth Edition; TOWRE-2 = Test of Word Reading Efficiency—Second Edition; NEPSY = NEuroPSYchology test; CCC-2 = Children’s Communication Checklist—Version 2; GCC = General Communication Composite; M = male; F = female; R = right; L = left.

^a^ Scaled scores with a standard mean of 10 and SD of 3.

^b^ Raw scores.

^c^ Scaled scores with a mean of 80 based on the sum of the eight subtests.

### Brain Morphometric Data

The DLD, TD, and HSL groups did not differ in the estimated total intracranial volume, extracted with FreeSurfer’s “aparcstats2table” command (measured in mm^3^; DLD *m* ± SEM = 1,323,593 ± 22,398; TD *m* ± SEM = 1,339,592 ± 15,868; HSL *m* ± SEM = 1,409,468 ± 34,784) or mean cortical thickness (measured in mm; DLD *m* ± SEM = 2.7 ± 0.012; TD *m* ± SEM = 2.7 ± 0.008; HSL *m* ± SEM = 2.7 ± 0.015). Children in the HSL group had significantly larger mean surface area values (measured in mm^2^; *m* ± SEM = 193,688 ± 4,240) than DLD children (*m* ± SEM = 182,028 ± 2,805), *F*(2, 156) = 3.31, *p* = 0.039. There were no differences between these groups and the TD group (*m* ± SEM = 186,051 ± 2,004).

We examined the influence of age and sex on our three cortical metrics (area, thickness, and volume) using the whole cohort (*n* = 156; i.e., including the HSL group). For surface area, there was no relationship with age and no effect of sex in any brain region. Regarding cortical thickness, a small number of regions exhibited a decrease in thickness with increasing age, including portions of the medial parietal and occipital cortex, left ventral occipital cortex, and right orbitofrontal cortex. Conversely, increases in thickness with age were detected in the posterior ventromedial frontal cortex and temporal poles (Supplementary Material Figure 2 and Table 1). We found that female participants had greater thickness than male participants in a small portion of the left fusiform cortex only (Supplementary Material Figure 3 and Table 2). For volume, we observed a significant negative correlation with age in a portion of the medial occipital cortex, with no effect of sex. We included age and sex as covariates in the analyses that compared DLD and TD children below as well as in the continuous analyses with the language factor.

### Whole-Brain Analysis of Cortical Morphology in DLD vs. TD Children

**Figure 1.**
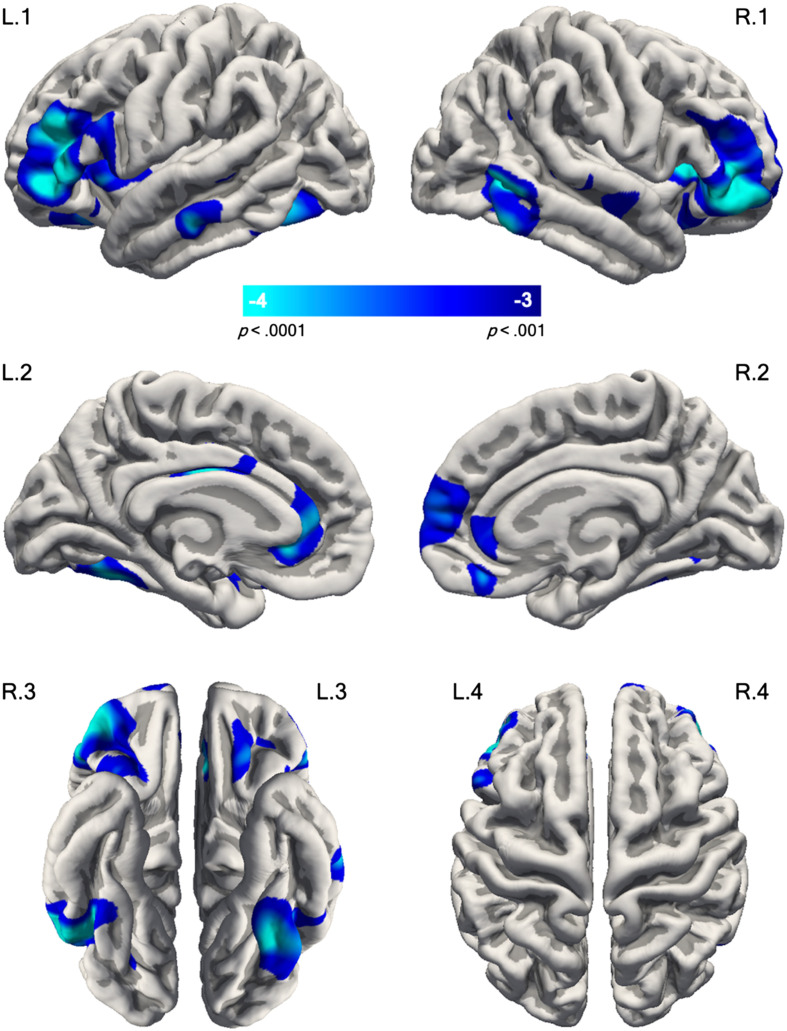
Cortical regions showing smaller surface area in DLD. Whole-brain statistical map illustrates group differences in surface area between DLD and TD children. The left and right hemispheres are shown on the left and right sides of the figure, respectively. The statistical map is overlaid on the pial surface of *fsaverage* and is displayed using an uncorrected vertex-wise threshold of *p* < 0.001 for visualisation purposes. The numbers inside the colour bar display the −log10(*p*), where *p* is the significance. The blue/cyan hues indicate the DLD < TD contrast. Cluster-corrected results are reported in the text. L.1/R.1 = lateral view; L.2/R.2 = medial view; L.3/R.3 = ventral view; L.4/R.4 = dorsal view.

Figure 1 depicts vertex-wise surface maps of brain regions where children with DLD had lower surface area than TD children. The DLD group had significantly smaller surface areas in symmetric portions of the anterior inferior frontal gyrus (pars triangularis and pars orbitalis) in both the left and right hemispheres. This difference extended posteriorly to pars opercularis in the left hemisphere and ventrally into the anterior insula bilaterally (Figure 1, L.1/L.4 and R.1/R.4). Additionally, smaller surface areas were observed in the lateral orbitofrontal cortex on the ventral surface of the left hemisphere (Figure 1, L.3). On the medial surface (Figure 1, L.2 and R.2), smaller surface area in DLD was observed in the rostral anterior cingulate cortex bilaterally, anterior medial superior frontal cortex on the right, and posterior cingulate cortex at the boundary with the corpus callosum on the left. Smaller surface area was also seen in DLD in the posterior temporal cortex and ventral occipitotemporal cortex at the level of the mid-fusiform gyrus bilaterally (Figure 1, L.1/L.3 and R.1/R.3).

Children with DLD also had lower grey matter volume in similar regions to those showing smaller surface area but only small clusters survived thresholding. These were in pars triangularis and the inferior temporal gyrus in the right hemisphere, and the middle temporal gyrus on the left. The anatomical location of statistical peaks using MNI305-space coordinates, along their corresponding *p* values and the extents of the cluster of voxels (in mm^2^) for surface area and grey matter volume are presented in Table 3. There were no group differences in cortical thickness.

**Table 3.** Brain regions showing significantly lower surface area and volume in the DLD group relative to the TD group

Cluster (peak)	Cluster-wise *p* value	Size	Centroid MNI305 coordinates		
			X	Y	Z
Surface area					
R superior frontal	0.0012	617	11	57	10
L pars orbitalis	0.0002	2269	−40	41	−6
R lateral orbitofrontal	0.0002	2212	29	25	3
L rostral anterior cingulate	0.0187	402	−61	25	−2
L pars opercularis	0.0077	471	−40	11	9
R posterior temporal	0.0002	1509	56	−53	−1
L fusiform	0.0002	1050	−33	−56	−16
Grey matter volume					
R pars triangularis	0.0002	452	47	36	−5
L middle temporal gyrus	0.0093	245	−59	−20	−22
R posterior temporal gyrus	0.0002	421	49	−57	−9

*Note*. Summary of significant clusters (*p* < 0.05, corrected) in the DLD < TD comparison for surface area and grey matter volume. The size of each cluster is reported in mm^2^ for surface area and mm^3^ for volume, along with the centroid MNI305 coordinates for Y (coronal), X (sagittal), and Z (axial) planes. Decimal values for the coordinates and cluster size were rounded to the nearest integer. Labels of peak location provided by FreeSurfer.

### Analyses of Cortical Morphometry and Language Proficiency in the Whole Cohort

We used cut-offs on norm-referenced tests of language to categorise participants into TD and DLD groups. This resulted in a group of 28 children with HSL who did not meet criteria for DLD due to performance above the cut-off. To test the relationship between language proficiency and our cortical metrics in the whole cohort, we used a language factor derived from the language and memory measures as a predictor of our three parameter values across the whole sample (*n* = 156), controlling for age and sex.

**Figure 2.**
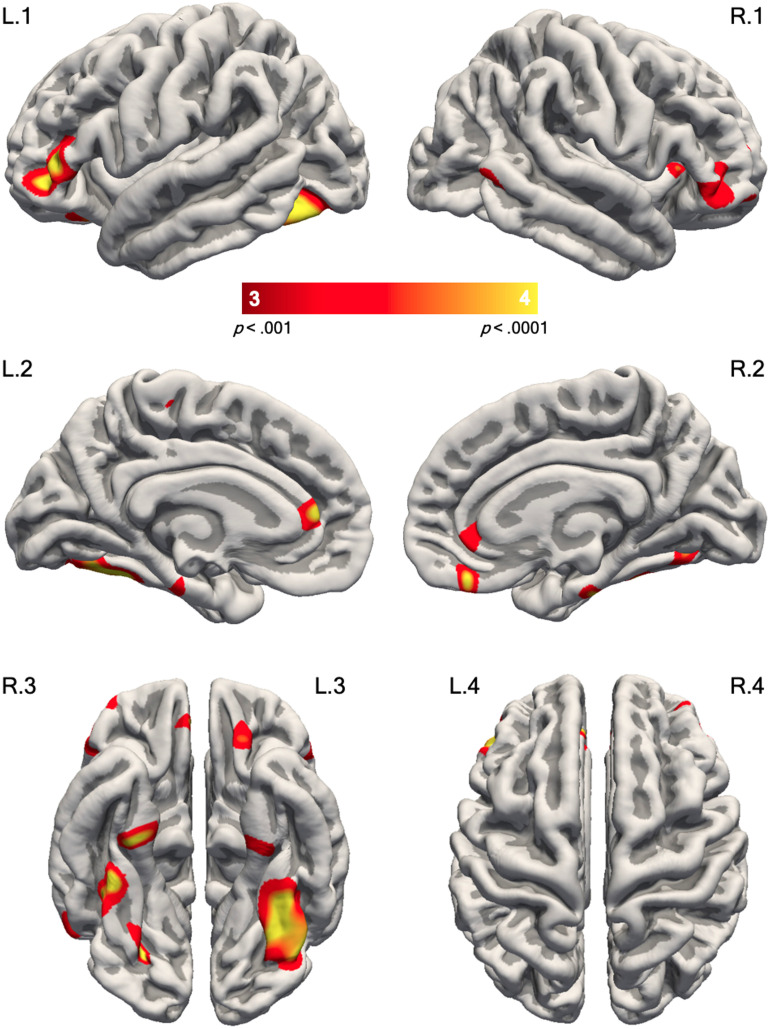
Continuous analysis of surface area and language proficiency scores in the whole cohort. The left and right hemispheres are shown on the left and right sides of the figure, respectively. Coloured significance maps are overlaid on the pial surface of *fsaverage* and displayed using an uncorrected vertex-wise threshold of *p* < 0.001 for visualisation purposes. Cluster-corrected results are reported in the text. The red/yellow hues indicate the positive correlation between surface area and language factors after removing the effects of age and sex. The numbers inside the colour bar display the −log10(*p*), where *p* is the significance. L.1/R.1 = lateral view; L.2/R.2 = medial view; L.3/R.3 = ventral view; L.4/R.4 = dorsal view.

We found significant relationships between the language factor and surface area (see Figure 2 and Table 4) in similar regions to those seen in our whole-brain group analysis (see Brain Morphometric Data); lower language ability was associated with smaller surface area. Significant positive correlations were observed in the pars triangularis of the inferior frontal gyri bilaterally, and this effect was stronger and more extensive on the left (Figure 2, L.1 and R.1). Significant positive correlations were also observed in the ventral occipitotemporal cortex at the level of the mid-fusiform gyrus (Figure 2, L.1/L.3 and R.1/R.3) also bilaterally but stronger on the left. The same pattern was observed in the continuous analysis of language factors and cortical volume, but results were less extensive and not as statistically robust as those seen in the analysis of surface area. There were no significant relationships between cortical thickness values and language proficiency.

**Table 4.** Brain regions showing positive correlation of surface area with language proficiency factors

Cluster (peak)	Cluster-wise *p* value	Size	Centroid MNI305 coordinates		
			X	Y	Z
Surface area					
L pars triangularis	0.0004	2212	−44	34	6
R pars triangularis	0.0183	419	41	40	2
L fusiform	0.0002	1385	−41	−61	−13
R fusiform	0.036	357	39	−40	−22

*Note*. Summary of significant clusters (*p* < 0.05, corrected) in the continuous analysis of surface area values and language factors. The size of each surface area cluster is reported in mm^2^, along the centroid MNI305 coordinates for Y (coronal), X (sagittal), and Z (axial) planes. Decimal values for the coordinates and cluster size were rounded to the nearest integer. Labels of peak location provided by FreeSurfer.

### Analysis of Structural Asymmetry in DLD vs. TD Children

For each of our 54 DLD and 74 TD participants, we generated surface area, cortical thickness, and grey matter volume asymmetry maps in which vertex-wise AQ values across the entire cortex were entered into a GLM with age and sex as nuisance variables. There were no differences between DLD and TD in the degree of structural asymmetry across the cortex in any of these three metrics. We re-ran the above analyses after excluding 19 participants (8 DLD, 11 TD) who reported being left-handed (using their left hand to write) and observed the same pattern of results for all three metrics (i.e., no significant group differences).

**Figure 3.**
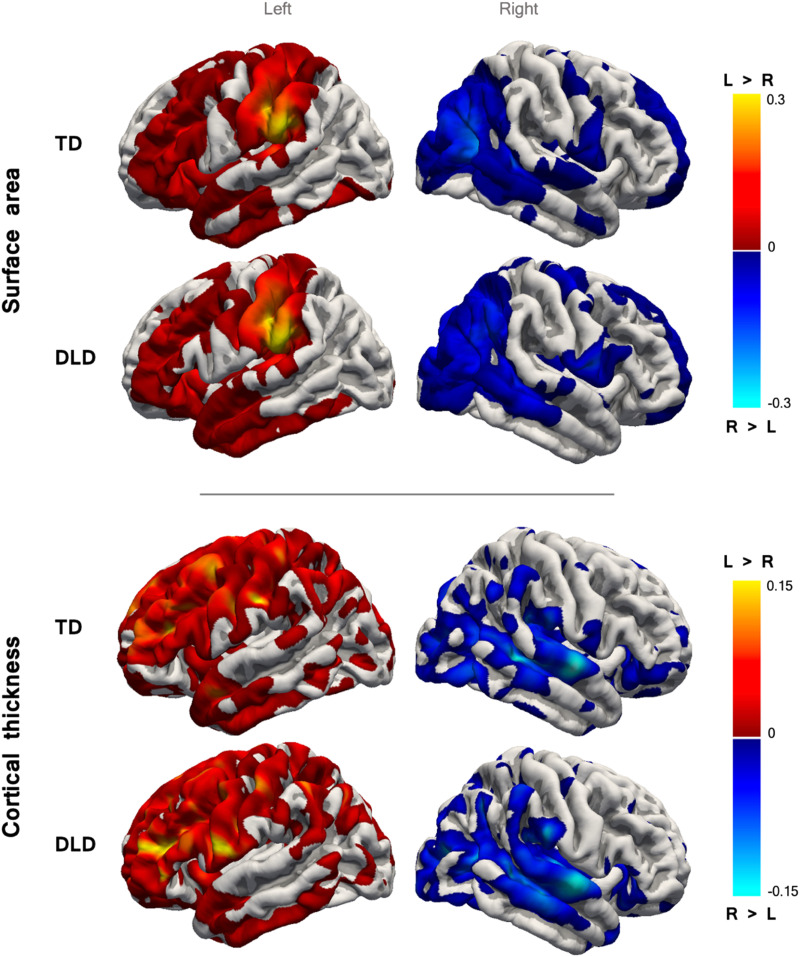
Cortical asymmetries for surface area and cortical thickness in DLD vs. TD children. Whole-brain maps show DLD and TD children’s mean asymmetry quotient (AQ) values for surface area and cortical thickness. The maps are overlaid on the pial surface (lateral view), and the left and right hemispheres are shown on the left and right sides of the figure, respectively. Red/yellow hues indicate left-greater-than-right asymmetry (L > R), and the blue/cyan hues indicate right-greater-than-left (R > L). L = left; R = right.

Figure 3 shows the mean AQ values indicating the degree of left- and right-ward structural asymmetry in surface area and cortical thickness, superimposed onto the left and right pial surfaces for DLD and TD participants. The patterns in both groups are very similar. In terms of surface area, both groups had a leftwards asymmetry (i.e., left-greater-than-right) in the frontal cortex and a rightwards asymmetry in the occipital lobe. The strongest leftward asymmetries in surface area were located in the supramarginal and post central gyri in both groups. For cortical thickness, there was a leftwards asymmetry in the frontal cortex, which extended posteriorly and dorsally to the parietal cortex. Frontally, the strongest left asymmetries in cortical thickness were seen in rostral middle frontal gyrus, extending posteriorly to pars triangularis and precentral gyrus. The middle to posterior portions of superior and middle temporal gyri showed a rightwards asymmetry in thickness.

## DISCUSSION

We investigated cortical morphology in a large, well-characterised sample of 156 children and adolescents aged 10–16 years with a range of language abilities including 54 children with DLD, 28 with HSL who did not meet criteria for DLD, and 74 age-matched TD. The DLD group had smaller surface area relative to the TD group in the inferior frontal gyrus extending to the anterior insula, in the posterior temporal cortex and the ventral occipitotemporal cortex, all bilaterally. Medially, portions of the anterior cingulate and superior frontal cortex also had smaller surface area in DLD. There were no group differences in cortical thickness. A similar pattern of results was obtained when using a continuous variable of language proficiency based on a factor analysis of scores on tests of language. This analysis included the children in the HSL group as well as DLD and TD. Poorer scores on the language factor were associated with smaller surface areas in the inferior frontal gyrus and ventral occipitotemporal cortex again bilaterally but with more extensive effects in the left hemisphere. As for the group analysis, there was no significant relationship between cortical thickness and language proficiency in any region.

Overall, in terms of anatomical location, these results are consistent with many of the previous findings of cortical differences in DLD reported using other methods and confirm our prediction that children with DLD would show differences in the inferior frontal gyrus. Our second prediction that these regions would also show atypical cortical asymmetry in DLD was not upheld: We found no significant differences in asymmetry between groups. The finding that surface area but not cortical thickness is affected in DLD provides new insights into the underlying processes of brain development in this disorder. To our knowledge, this is the first study in the DLD literature to focus on these separate morphological traits of the cortex. Below we discuss the implications of these findings with relevance to the relationship between cortical structure and function in the context of a developing brain.

### Inferior Frontal Cortex Anomalies in DLD

The left inferior frontal gyrus consists of a mosaic of subregions implicated in phonological, semantic, and syntactic processing (e.g., see Vigneau et al., 2006). We found smaller surface area in our DLD cohort in all three subregions of this structure, namely pars orbitalis, triangularis, and opercularis, bilaterally. The results of the continuous analysis with the language factor showed differences focused on the pars triangularis extending anteriorly to the orbitalis and these effects were more extensive on the left than the right. There is only one other study which reported structural abnormality in pars orbitalis in DLD (Lee et al., 2020), but this difference was a volumetric *enlargement* in the right hemisphere and the DLD groups were older on average, and with fewer participants than ours (*m* age = 22.42 and 16.96 yr, *n* = 14 and 19 in cohorts 1 and 2, respectively; see Table 1). Two other studies reported volume enlargement in the left inferior frontal gyrus in DLD in a roughly similar age range to ours (Badcock et al., 2012; *m* age = 13.5 yr; *n* = 10) and more specifically, in pars triangularis (Kurth et al., 2018, *m* age = 10.21 yr; *n* = 13); but again the sample sizes were much smaller than ours. The only study that reported lower surface area in pars triangularis (measured through manual tracing with a mouse-operated cursor) was by Gauger et al. (1997) in a slightly younger and much smaller sample (*m* age = 9.1 yr; *n* = 11).

Pars orbitalis has been associated with an executive function role in semantic cognition tasks in adults (Krieger-Redwood et al., 2015) as well as narrative comprehension (Babajani-Feremi, 2017; Mar, 2011). In children, left pars orbitalis and triangularis are especially implicated in semantic processing (Skeide & Friederici, 2016). It has been argued that children rely more on these regions to process meaning from syntactically complicated sentences while their grammatical skills are still developing (Enge et al., 2021). Pars opercularis plays a critical role in syntactic ability as children’s grammatical abilities become more mature towards adulthood (Friederici, 2017; Hagoort, 2014; Skeide & Friederici, 2016). In adults, noninvasive brain stimulation over pars triangularis and pars opercularis selectively impairs semantic and phonological judgements, respectively (Gough et al., 2005). The strong connections between pars opercularis and the motor representations for the articulators are consistent with its known role in spoken word production (Flinker et al., 2015). Given the functional significance of these cortical regions for language and their substantial connectivity to other brain areas (Bulut, 2022), the anomalous morphology of the inferior frontal cortex in children with DLD is consistent with the behavioural profile of impaired language processing that characterises this population.

### Ventral Occipitotemporal Anomalies in DLD

The region showing smaller surface area in the left hemisphere in the DLD group analysis was very much centred on the mid-fusiform gyrus. The same region showed significant correlation between surface area and language proficiency when the whole cohort was analysed. This portion of cortex is considered a hub for graphophonological word processing (Cohen & Dehaene, 2004). Several studies provided evidence for the involvement of this region in long-term memory representations of visual word forms. Functional and structural abnormalities in this region are reported in children with dyslexia (Kronbichler & Kronbichler, 2018; Paulesu et al., 2014). DLD and dyslexia are neurodevelopmental disorders that frequently co-occur (Bishop & Snowling, 2004) and possibly have a shared aetiology (Ramus et al., 2013). It is therefore not surprising to see overlapping structural abnormalities in both disorders. In fact, prior surface-based morphometric studies have found smaller area in both the inferior frontal gyrus and fusiform gyrus in individuals with dyslexia (Frye et al., 2010).

Good oral language provides the foundation on which later literacy skills develop (Botting et al., 2006). This is supported by studies that found higher risk of reading problems in children with a history of oral language impairments (Snowling et al., 2000). Deficit in spoken language comprehension is a predictor of later reading difficulties in children with DLD (Tallal et al., 1988), likely reflecting the influence of early phonological skills on the development of literacy (Hulme et al., 2015). Cortical differences in the mid-fusiform gyrus in DLD could therefore be attributed to an underdeveloped neural network of skillful reading, perhaps due to lower print exposure.

### No Differences in Cortical Asymmetry Associated With DLD

Previous structural asymmetry studies linked structural deviation in hemispheric dominance to language impairments (Jernigan et al., 1991; Plante, 1991; Plante et al., 1989, 1991), a finding we failed to replicate. In accordance with a lack of difference in cortical asymmetry, previous work in DLD has also failed to find evidence to support altered lateralisation of function. For example, a previous functional MRI investigation of children with DLD found no difference in language lateralisation compared with TD during verb generation (Krishnan et al., 2021). Additionally, in a well-powered investigation of a sample of twin children with over 100 cases of DLD, Wilson and Bishop (2018) used transcranial Doppler ultrasonography to show that there were no differences between school-age DLD and TD children on language lateralisation. In fact, they even found a greater incidence of right laterality for language in TD children, suggesting that an “atypical” pattern of language lateralisation is not necessarily at odds with developing typical language skills (Wilson & Bishop, 2018). On the basis of both our structural findings reported here and previous functional findings, we conclude that there is no evidence for consistently altered language lateralisation or cerebral asymmetry in children with DLD. The inconsistency in current results compared with past findings might either reflect a lack of sufficient statistical power due to the small number of participants, or that previous findings are false positives.

### Cortical Surface Area and Cortical Thickness Development

The differences found in DLD in this study were limited to cortical surface area and grey matter volume, with differences in the latter being neither extensive nor as statistically robust as those seen in the analysis of surface area. There were no group differences in cortical thickness. Since volume is the product of area and thickness, the volume differences were most likely driven by the differences in surface area.

Postnatally, surface area increases over the first decade of life and then plateaus during adolescence (Raznahan et al., 2011; Tamnes et al., 2017). In contrast, there are major changes in cortical thickness in the first two years, followed by very slow linear decreases with age that continue across the second decade (Amlien et al., 2016; Lyall et al., 2015). It is worth noting that in our study of children and adolescents aged 10–16 years, we found no relationship between age and surface area and only a handful of brain areas showed a reduction in cortical thickness across this age range.

Cafiero and colleagues (2019) proposed that areal expansion of the cortex during development is related to the myelination of cortico-cortical fibres. Notably, a recent quantitative MRI study provides a clue to differences in the underlying microstructure of the cortex in DLD (Krishnan et al., 2022). Using multiparametric mapping, these authors found lower levels of myelin, indexed through lower magnetisation transfer saturation values, in DLD in several regions of the left hemisphere’s speech and language network, including in pars opercularis, insula, and several parts of the temporal lobe. In addition, lower mean global values in longitudinal relaxation rate (R1, another myelin marker) were reported for children with DLD relative to TD, reflecting potentially widespread differences in myelin across cortical and subcortical grey matter bilaterally (Krishnan et al., 2022).

Depending on whether cortical differences in DLD are viewed as abnormal or delayed in maturation, lower surface area might be related to any number of the aforementioned neurodevelopmental processes. It might be the case that the affected regions in DLD have not undergone typical processes of neurogenesis prenatally. Surface area differences between DLD and TD could indeed be driven by genetic risk factors. Human genome-wide association studies have identified many loci for common genetic variants that explain variance in regional surface area and cortical thickness (Grasby et al., 2020). The genetic influences seen were stronger for surface area than for cortical thickness. Moreover, Beelen and colleagues (2019) found that surface area anomalies in the temporal cortex of a cohort of pre-readers related to a family risk for dyslexia, regardless of their later reading ability. Relatedly, in a large genome-wide association study, shared genetic factors were identified as contributing to surface area of the superior temporal sulcus and performance on measures of reading and language, indicating links at the molecular level to variation in these traits (Eising et al., 2022).

While surface area differences in DLD might reflect a causal link to as yet unknown genetic disruptions, it cannot be ruled out whether the observed cortical differences are a *consequence* of language learning difficulties. In other words, plastic changes in the cortical areas involved in typical language processing may not have occurred at the same time or rate in the children with DLD because of their different language-learning experience. The latter argument could explain the strongly left-lateralised pattern of less cortical myelin indexed by magnetisation transfer saturation in DLD in the study by Krishnan and colleagues (2022) compared with the lower R1 myelin marker that affected the brain globally. The language learning impairment seen in our study in children with DLD might have delayed the typical pattern of maturation of cortical areas, through a failure in specialisation of function in circuits involving these regions. Teasing apart the contributions of genetic risk factors and experience-dependent plasticity is an important next step in DLD neuroimaging work, which will require detailed longitudinal investigation.

### Limitations and Future Directions

This study is limited by its cross-sectional nature, which prevents us from discerning whether the observed cortical differences are the cause of DLD or a consequence of having a language disorder. Longitudinal studies of large DLD cohorts in which children are followed over time could shed light on this question, while also having sufficient power to identify statistically robust differences in cortical metrics. Longitudinal investigations could also enhance our understanding of how these structural differences might affect specific aspects of function. Pursuing this question is especially important for guiding clinically oriented studies that attempt to answer whether cortical abnormalities could be targeted through behavioural interventions early on, while also gaining insight on the underlying mechanisms of cortical plasticity. A longitudinal comparison could also help to understand whether the trajectories of cortical development are abnormal (i.e., deviant from TD children at all time points) or delayed (i.e., resemble those of TD children at a younger age) in DLD.

DLD has a complex polygenic and multifactorial aetiology (Mountford et al., 2022), and the interaction of multiple genes with environmental influences give rise to considerable heterogeneity in the phenotypic manifestations of this disorder. In other words, the language deficit profiles associated with DLD commonly differ on a case-by-case basis. This variability increases when DLD is viewed within the dynamic course of development, with evidence showing that the language phenotype does not remain the same across different age ranges (Botting, 2005). Another point is that developmental disorders rarely occur in isolation. Despite excluding those with co-occurring diagnoses such as ASD and ADHD in the current study, participants with a range of other neurodevelopmental conditions in addition to DLD might have been grouped together. Overall, considering this heterogeneity and the (non)linear and region-specific maturation trajectories of the cortex, it might be that current methods of group analysis are simply not able to account for varying profiles of language disorder throughout development. A promising line of research is to conduct large-scale studies of children with multiple diagnoses using a transdiagnostic approach, to probe whether specific relationships between neural measures and behaviour could be established (Siugzdaite et al., 2020).

Lastly, it is worth noting that puberty is another important factor associated with brain development during adolescence which further complicates attempts to find answers to the questions posed throughout this section. Measuring puberty through self-report can be unreliable and hormonal testing brings practical challenges (see Herting & Sowell, 2017, for a review). Even though we acknowledge the importance of pubertal influences on both behavioural and brain changes in this period, accounting for these effects was beyond the scope of this study. Longitudinal studies with several time points starting earlier in development and with even larger sample sizes would be required to capture the cortical maturation trajectories in relation to language and explore their interactions with physical and hormonal markers of puberty. Such studies could also shed light on whether the findings presented here are unique to the age period studied or are present earlier in development.

## CONCLUSION

Investigating the neural correlates of DLD is particularly challenging given the developmental nature of the disorder and the heterogeneity associated with it. In our investigation of a large and well-characterised DLD neuroimaging dataset, we found that the main cortical difference between TD children and those with DLD was in surface area, where a bilateral, and highly symmetric pattern of smaller area was seen in several brain regions in DLD. Notably, these regions survived stringent correction for multiple comparisons. Large DLD cohorts are necessary to identify statistically robust differences in cortical metrics. Future longitudinal work is required to explore how these cortical differences change during development (e.g., whether their trajectory is delayed or abnormal), whether DLD is the cause or consequence of the observed differences, and what their relationship is to function. Clinically oriented studies are also required to assess whether cortical abnormalities could be targeted through training using behavioural interventions early in development.

## DATA AND CODE AVAILABILITY STATEMENT

All statistical analyses were conducted using MATLAB R2022b (MathWorks, 2022) and the R programming language (R Core Team, 2022). The anonymised neuropsychological scores and codes supporting the findings of this study are openly available on the Open Science Framework (https://osf.io/wmkrq/). Statistical maps of group differences can be viewed on NeuroVault (https://identifiers.org/neurovault.collection:13977).

## ACKNOWLEDGMENTS

We are very grateful to our participants and their families for dedicating their time to take part in our study. Without their contribution, this research would not have been possible. We would also like to acknowledge the various individuals and organisations that helped us with recruitment (https://boldstudy.wordpress.com/acknowledgements/). We thank Professor Dorothy Bishop for her encouragement and support. We thank Dr. Caroline Nettekoven for her assistance with data curation. We also thank members of the Wellcome Centre for Integrative Neuroimaging, especially the MRI team at the Oxford Centre for Human Brain Activity: Sebastian Rieger, Juliet Semple, Nicky Aikin, Nicola Filippini, Eniko Zsoldos, and Emily Hinson. We also extend our gratitude to Zhiqiang Sha and Clyde Francks, members of the Language and Genetics Department at Max Planck Institute for Psycholinguistics, for sharing their template scripts for the asymmetry analyses.

## FUNDING INFORMATION

The Oxford Brain Organisation in Language Development or OxBOLD study was funded by the Medical Research Council MR/P024149/1 to Kate E. Watkins, and supported by the NIHR Oxford Health Biomedical Research Centre. The Wellcome Centre for Integrative Neuroimaging is supported by core funding from the Wellcome Trust (203139/Z/16/Z). For the purpose of open access, the authors have applied a CC BY public copyright license to any Author Accepted Manuscript version arising from this submission.

## AUTHOR CONTRIBUTIONS

**Nilgoun Bahar**: Conceptualization: Lead; Formal analysis: Lead; Visualization: Lead; Writing – original draft: Lead. **Gabriel J. Cler**: Formal analysis: Supporting; Investigation: Supporting; Project administration: Supporting; Writing – review & editing: Supporting. **Saloni Krishnan:** Funding acquisition: Supporting; Investigations: Lead; Project administration: Lead; Writing – review & editing: Supporting. **Salomi S. Asaridou**: Investigation: Supporting; Project administration: Supporting; Writing – review & editing: Supporting. **Harriet J. Smith**: Investigation: Supporting; Writing – review & editing: Supporting. **Hanna E. Willis**: Investigation: Supporting; Writing – review & editing: Supporting. **Máiréad P. Healy**: Investigation: Supporting; Writing – review & editing: Supporting. **Kate E. Watkins**: Conceptualization: Lead; Formal analysis: Supporting; Funding acquisition: Lead; Supervision: Lead; Visualization: Supporting; Writing – review & editing: Lead.

## Supplementary Material


