# Supplementary material for: Differences in Cortical Surface Area in Developmental Language Disorder
Source: Neurobiol Lang (Camb). 2024 Jun 3;5(2):288–314. doi: 10.1162/nol_a_00127 (PMC11093399; doi:10.1162/nol_a_00127)
Supplement: Supplementary file 1 [file nol-5-2-288-s001.pdf]

Supplementary Material

Differences in cortical surface area in developmental language disorder

Nilgoun Bahar[1], Gabriel J Cler[1,2], Saloni Krishnan[1,3], Salomi S Asaridou[1], Harriet J Smith[1,4], Hanna E Willis[1,5], Máiréad P Healy[1,6], Kate E Watkins[1]

[1] Department of Experimental Psychology & Wellcome Trust Centre for Integrative Neuroimaging, University of Oxford, Oxford, UK

[2] Department of Speech & Hearing Sciences, University of Washington, Seattle, USA

[3] Department of Psychology, Royal Holloway, University of London, Egham Hill, Surrey, UK

[4] MRC Cognition & Brain Sciences Unit, University of Cambridge, Cambridge, UK

[5] Nuffield Department of Clinical Neuroscience, University of Oxford, Oxford, UK

[6] Department of Psychology, University of Cambridge, Cambridge, UK

Corresponding author

Nilgoun Bahar, Department of Experimental Psychology, Oxford OX2 6GG, United Kingdom.

Email: nilgoun.bahar@psy.ox.ac.uk

Contents

Supplementary Figures 1-3

Supplementary Tables 1-2

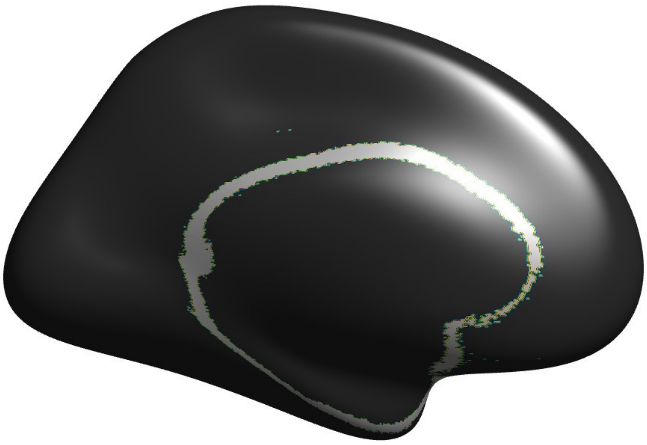

Supplementary Figure 1.

The exclusion mask used in the structural asymmetry analysis. The mask comprised vertices at the boundary between cortical and subcortical structures with extreme |AQ| values > 1 in at least one quarter of our participants. This left 157,992 vertex-wise asymmetry measures per individual spanning the rest of the cortex. |AQ| values above one possibly indicate a resampling artifact to the *fsaverage_sym* template.

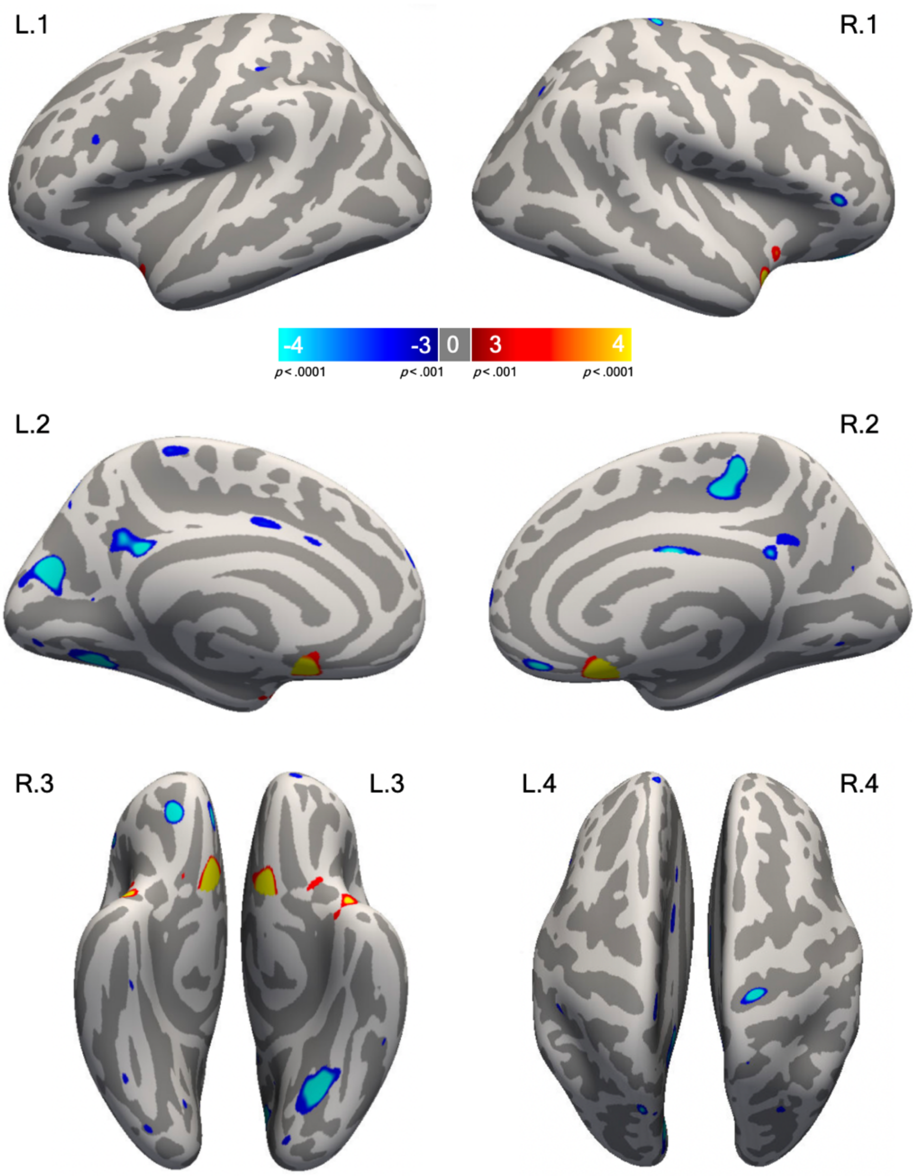

Supplementary Figure 2.

Whole-brain significance map of age effects on cortical thickness across all participants, depicted on the inflated surface. Results are displayed using an uncorrected vertex-wise threshold $p < .001$. Positive correlations are shown in red/yellow and negative ones in blue/cyan. Cluster-corrected results are reported Supplementary Table 1. L.1/R.1 = lateral view; L.2/R.2 = medial view; L.3/R.3 = ventral view; L.4/R.4 = dorsal view.

Supplementary Table 1.

Summary of significant clusters for the change in (A) cortical thickness and (B) grey matter volume with age across all participants. The size in mm^2 and centroid MNI305 coordinates are reported for significant clusters ($p < .05$, corrected). The coordinate decimals and cluster sizes were rounded to integer values.

**Cluster (peak)**	**Cluster-wise *p* value**	**Size**	**Centroid MNI305 Coordinates**		
			**X**	**Y**	**Z**
**(A) Cortical thickness**					
Positive correlations					
L temporal pole	.0431	169	-34	5	-31
R medial orbitofrontal	.0286	186	6	19	-17
R superior temporal	.0476	164	41	4	-25
Negative correlations					
L isthmus cingulate	.0201	197	-5	-45	30
L fusiform	.0002	452	-30	-64	-6
L cuneus	.0002	486	-12	-75	19
R paracentral	.0084	227	9	-33	52
**(B) Grey matter volume**					
L cuneus (negative correlation)	.0008	362	-10	-68	10

Labels of peak location provided by FreeSurfer.

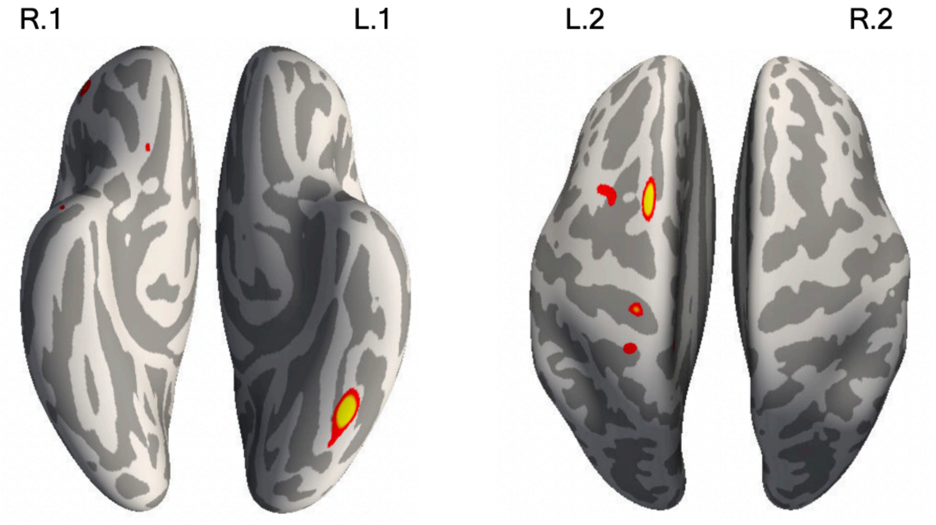

Supplementary Figure 3.

Whole-brain significance map of sex effects on cortical thickness across all participants, depicted on the inflated surface. Results are displayed using an uncorrected vertex-wise threshold $p < .001$. Areas where cortical thickness was greater in girls compared with boys are shown in red/yellow. There were no areas where cortical thickness was greater in boys compared with girls. Only the cluster in the left fusiform gyrus survived correction. See Supplementary Table 2. (L.1/R.1 = lateral view; L.2/R.2 = medial view; L.1/R.1 = ventral view; L.2/R.2 = dorsal view.

Supplementary Table 2.

Summary of significant clusters in the Males < Females comparison for cortical thickness across all participants. The size in mm^2 and centroid MNI305 coordinates are reported for significant clusters ($p < .05$, corrected). The coordinate decimals and cluster sizes were rounded to integer values.

Cluster (peak)	Cluster-wise *p* value	Size	Centroid MNI305 Coordinates		
			X	Y	Z
**Cortical thickness**					
L fusiform	.0074	234	-42	-59	-20

Labels of peak location provided by FreeSurfer.
